# Exploring Spillover Effects for COVID-19 Cascade Prediction

**DOI:** 10.3390/e24020222

**Published:** 2022-01-31

**Authors:** Ninghan Chen, Xihui Chen, Zhiqiang Zhong, Jun Pang

**Affiliations:** 1 Faculty of Sciences, Technology and Medicine, University of Luxembourg, L-4364 Esch-sur-Alzette, Luxembourg; ninghan.chen@uni.lu (N.C.); zhiqiang.zhong@uni.lu (Z.Z.); 2 Interdisciplinary Centre for Security, Reliability and Trust, University of Luxembourg, L-4364 Esch-sur-Alzette, Luxembourg; xihui.chen@uni.lu

**Keywords:** cascade prediction, information diffusion, COVID-19, graph neural networks, spillover effects, Twitter

## Abstract

An information outbreak occurs on social media along with the COVID-19 pandemic and leads to an *infodemic*. Predicting the popularity of online content, known as *cascade prediction*, allows for not only catching in advance information that deserves attention, but also identifying false information that will widely spread and require quick response to mitigate its negative impact. Among the various information diffusion patterns leveraged in previous works, the *spillover effect* of the information exposed to users on their decisions to participate in diffusing certain information has not been studied. In this paper, we focus on the diffusion of information related to COVID-19 preventive measures due to its special role in consolidating public efforts to slow down the spread of the virus. Through our collected Twitter dataset, we validate the existence of the spillover effects. Building on this finding, we propose extensions to three cascade prediction methods based on Graph Neural Networks (GNNs). Experiments conducted on our dataset demonstrated that the use of the identified spillover effects significantly improves the state-of-the-art GNN methods in predicting the popularity of not only preventive measure messages, but also other COVID-19 messages.

## 1. Introduction

The outbreak of the COVID-19 pandemic leads to an *infodemic*, which is partially attributed to the outbreak of information on major online social networks (OSNs), including Twitter, Facebook, Instagram, and YouTube [1]. Due to physical isolation and social distancing, people spent much more time on OSNs, engaging in expressing opinions, catching up-to-the-minute development of the pandemic and even looking for medical support and knowledge to ease mental depression and seek psychological comfort. This new change of information perception makes OSNs an essential communication channel for healthcare departments and medical staff to disseminate official policies and professional advice promoting effective measures to prevent the spread of the COVID-19 virus, e.g., wearing masks, vaccination and social distancing. Meanwhile, misinformation and false news also take advantage of OSNs to spread with unprecedented speed and volume and result in risk-taking behaviours that will harm public health. As a consequence, this information explosion on OSNs impedes the efficacy of pandemic response and increases public confusion about who and what *preventive measures* to trust [2]. To combat theinfodemic, one widely accepted approach is known as *cascade prediction*, the purpose of which is to learn the popularity of messages according to its early adopters. Accurate prediction can help the public catch information deserving special attention, and assist healthcare departments with identifying misinformation that requires fast response to control its negative impact.

Research on cascade prediction has been sustained, with a large number of prediction models developed. Earlier models rely on hand-crafted features extracted from demographic profiles of early adopters [3,4] or social graphs composed of early adopters and their relationships [5]. The recent advances of deep learning lead to models that can automatically learn useful features, encoded as a low-dimensional representation of available evidence that can be intuitively interpreted as most related features [6,7]. In particular, the application of graph neural networks (GNN) allows for capturing the features of nodes’ neighbourhoods and simulating information cascading over social networks [8].

In spite of the various diffusion patterns exploited, previous studies have not considered the *spillover effect* of a user’s exposed information on his/her behaviour of forwarding certain types of messages and becoming part of their diffusion, which we call *info-exposure spillover effect* for short. Spillover effects have become a commonly adopted theory in studying the impact of certain information on the opinions and behaviour changes of information consumers. For example, studies of political attitudes have found that exposure to scandals about some candidates may have negative spillover effects on the public’s trust in other politicians [9,10]. We say a user is *exposed* to a message if the user posts the message or perceives it from his/her friends on social media. In this paper, we adopt the original definition of a behaviour spillover effect which intuitively means *“the observable and causal effect that a change in one behaviour has on a different, subsequent behaviour”* [11]. According to this definition, the info-exposure spillover effect studied in this paper can be interpreted as the impact of the information a user perceived from the social media on his/her behaviour of forwarding a COVID-19 related post received from his/her friends.

We hypothesise the existence of this info-exposure spillover effect according to the previous studies related to the COVID-19 pandemic. Park et al. [12] demonstrated that information with a medically oriented thematic framework has a wider spillover effect on COVID-19 issues in a Twitter context. Racist information is found to have spillover effects on the mistrust of medical system [2] and thus harm public trust in the information released by these systems.

In this paper, we focus on the diffusion of messages related to COVID-19 preventive measures considering their importance in slowing down virus transmission and protecting public health. After the outbreak of the pandemic, the topics of information to which social media users are exposed have experienced subtle changes. Some of these changes may subsequently lead to the changes of their intention to forward messages concerning preventive measures. For example, tweets about unemployment or loneliness may make a user who reads them perceive the severity of the pandemic and thus become more likely to retweet tweets about staying at home.

**Our contributions.** We collected a dataset from Twitter which contains both users’ posted messages and their social relations with others. With this dataset, we successfully validated the existence of the info-exposure spillover effect of users’ exposed messages on their decisions to retweet messages related to preventive measures. Specifically, we take into account all the messages exposed to users, regardless of whether they were related to COVID-19 or not. We observed that, although all messages present certain a level of spillover effects on retweeting preventive messages, those related to COVID-19 have stronger impacts. This motivates us to extend existing state-of-the-art cascade prediction models by taking into account info-exposure spillover effects. Through comprehensive experimental evaluation on our dataset, we show that our extended models can increase the cascade prediction performance up to 23.84% in COVID-19 messages related to preventive measures. In order to attest whether info-exposure spillover effects also exist for other messages, we also run the extended models to predict the size of cascades of general messages concerning COVID-19 but not related to preventive measures. The results show an obvious increase in accuracy due to the use of the info-exposure spillover effect.

## 2. Related Work

**Cascade prediction.** Cascade prediction becomes attractive after studies reveal that some key properties of information cascades can be predicted [3,13]. In general, the cascade prediction methods can be divided into two classes: macro-level prediction and micro-level prediction. Micro-level prediction aims to predict users who will be activated during the information diffusion, while macro-level cascade prediction directly calculates the final size of targeted cascades.

The idea of most micro-level methods are based on the Independent Cascade model (IC) [14], which calculates the probability of influence between every pair of users [15]. These methods rely on a number of assumptions that overly simplify the real situation such as the complete observation of diffusion processes [16]. Although Deepinf [17] uses an end-to-end deep learning method to overcome such assumptions, micro-level methods generally do not perform well in predicting cascade future size as they require simulating the entire diffusion process. In this paper, as our target is popularity prediction, we opt for macro-level methods.

Macro-level prediction methods can be divided into three categories as a result of technological evolution, i.e., statistical prediction model, machine learning-based methods and deep learning-based methods. The development of macro-level prediction started with statistical models such as SEISMIC [18] and Weibull [13]. Then, the advancements of machine learning led to methods using manually designed features extracted from text content, temporal and demographic information, and network structure [3,4,13]. Deep learning-based methods overcome the deficiency of machine learning-based methods of constructing manual features and capture effective features automatically. DeepCas [19] and DeepHawkes [20] use Recurrent Neural Networks (RNNs) to capture cascading sequences in place of manually designed features. However, RNNs are limited in capturing structural information. This limitation is addressed by graph neural networks (GNNs) [21]. Intuitively, GNNs update the representation of each node by recursively aggregating the representations of its neighbours. In this way, the iterated node representation summarises both structural and representation information in neighbourhoods. CasCN [22] utilises a dynamic Graph Convolutional Network (GCN) to learn the structural information of the cascade. CoupledGNN [8] (CGNN) effectively addresses cascade prediction with two GNNs, capturing the cascading effect which indicates that the activation of one user will successively trigger its neighbours.

Although deep learning-based methods have achieved relatively good results in cascade prediction, little research has been conducted to incorporate textual content into cascade prediction. Users’ textual posts, as an important part of social media, may contain information that are related to users’ decision to participate in diffusion of certain messages. Thus, we narrow the focus in this article to macro-level cascade prediction by extending the existing models to leverage online textual content on social media.

**Spillover effects.** The spillover effect has been widely used to study the impact of information on the information consumers’ opinion and behaviour [9,10,23,24]. Spillover effects can be interpreted and explained in various ways. We identify two main typologies in the literature, namely *behavioural spillover effects* and *affective spillover effects*.

The former interprets spillover effects as implicit ideas people build up that two things are connected, regardless of whether they are in the same context or across different contexts [10]. For instance, Sikorski explained the damage of the public’s trust in politicians following scandals of candidates as a behavioural spillover effect [9]. Other examples include the impact of religious activities on political orientation [25], and imposition of extra congestion charges on environmental behaviour changes in situations irrelevant to traffic [23]. The latter studies how affective responses (e.g., emotions such as happiness and anger) triggered by certain information affect human behaviour, usually based on the ‘feelings-as-information’ model [24], Schwarz et al. found that anger triggered by other information may have negative effects on people’s political attitudes [26]. Yegiyan discovered that the emotional feelings caused by film clips shown before commercial advertisements may affect audience’s product preference [27].

Based on these previous studies, we make our hypothesis that, during the COVID-19 pandemic, the information exposed to an individual may have spillover impacts on his/her behaviour of retweeting messages. In our validation (see Section 5), we consider both behavioural or affective spillover effects. To capture our info-exposure spillover effect, we do not explicitly distinguish these two typologies and profit from the power of deep learning to automatically learn the features of exposed messages that have spillover effects.

## 3. Preliminaries

### 3.1. Problem Definition

In this section, we give the formal definition of the popularity prediction problem studied in this paper which takes into account both social relations and online textual contents.

We use graph G=(V,E) to denote the social network where V is the set of nodes representing users and E⊆V×V is the set of edges indicating the relationships between users. For each v∈V, given a time period, we use $M_{v}$ to denote the messages posted by the user corresponding to *v*, and M to denote the set of all messages, i.e., $M=\cup_{v\in V}M_{v}$. In the rest of the paper, we will misuse the notions of users and nodes whenever it is clear from the context.

When a message *m* is firstly posted by a user, it will be perceived by the user’s followers who might adopt the message and relay the message. This cascading process will continue on the social network until no further sharing occurs. We denote the observed diffusion cascade of *m* at time *t* by $C_{m}^{t}=\left\{v_{1},v_{2},\ldots ,v_{n_{t}^{m}}\right\}$ ($v_{i}\in V$ for $1\leq i\leq n_{t}^{m}$), i.e., the set of users who had adopted *m* before *t*. Note that $n_{t}^{m}$ is the number of the adopters of *m* at *t*. Compared to the previous works, we take into account the online textual messages posted by users in addition to the social network. This leads to the following definition.

**Definition** **1** (Online textual content-aware cascade prediction). *Given the cascade of message m at time t (i.e., $C_{m}^{t}$), social network G=(V,E) and the messages posted by users in V, i.e., $\forall_{v\in V}M_{v}$, the problem is to predict the final popularity of m at time ∞, i.e., $n_{\infty }^{m}$.*

As mentioned previously, we focus on the diffusion of the messages related to COVID-19 preventive measures, although we will also show the effectiveness of our extended models in predicting the popularity of other general messages. To integrate the online textual messages, i.e., M, in solving the problem, we will make use of the info-exposure spillover effects of messages exposed to users on their decision to relay preventive measure-related messages.

### 3.2. General Framework of GNNs

The purpose of graph neural networks (GNN) is to learn node representations of a graph. Compared to graph embedding works such as node2vec [28] and DeepWalk [29], one advantage of GNN is that it allows for integrating node attributes into the learning process. GNN is implemented with multiple layers. At each layer, a node’s representation is updated by combining the representations of their neighbours calculated in the previous layer. Intuitively, a *k*-layer GNN calculates a representation for each node by combining the attributes of the nodes within *k* hops. We adopt the formal definition in [21] and give the general definition of the *ℓ*-th layer (ℓ≤k) for a node v∈V as follows:

$$\begin{array}{ccc} a_{v}^{\ell } & = & \mathrm{Aggregate}(\left\{h_{u}^{\ell }:u\in N\left(v\right)\right\})\\ h_{v}^{\ell +1} & = & \mathrm{Combine}(h_{v}^{\ell },a_{v}^{\ell }) \end{array}$$

where $h_{v}^{\ell }$ is the representation vector of node *v* at the *ℓ*-th layer and N(v) denotes the set of neighbours of node *v*. Function Aggregate and Combine are instantiated according to the application scenarios so as to capture the most useful features of nodes’ neighbourhoods. This leads to the large number of GNN variants in the literature. With the representation vector of every node at the *k*-th layer, the representation of the graph G can thus be calculated by a function as follows: $h_{G}=\mathrm{Readout}\left(\left\{h_{v}^{k}:v\in V\right\}\right)$. The Readout function can be simply implemented as the mean of nodes’ vectors or other complex pooling functions depending on the specific requirements of scenarios in practice.

## 4. Data Collection and Pre-Processing

Twitter, one of the most prominent online social media platforms, has been used extensively during the COVID-19 pandemic. We select the Greater Region (GR) (The Greater Region of Luxembourg is composed of the Grand Duchy of Luxembourg, Wallonia, Saarland, Lorraine, Rhineland-Palatinate and the German-speaking community of Belgium.), a region with a population of high mobility, as the targeted area. This section presents how we built the dataset, constructed the cascades and built the social graph for our analysis and experiments.

### 4.1. Data Collection

In our dataset, we collected two types of data: (i) All the tweets posted or re-tweeted by GR users; (ii) the social networks of GR users recording their following relationships. In what follows, we elaborate the three steps we followed to gather these data.

- **Step 1. Tweet collection.** In this step, we collect a set of seed users in GR who actively participated in COVID-19 discussions and the tweets they originally posted or retweeted. Instead of searching by keywords, we refer to a publicly available dataset which contains the IDs of COVID-19 related tweets [30]. We extracted the tweet IDs posted between 22 January 2020 and 18 July 2020. This period covers the first wave of the pandemic. Through these tweet IDs, we download their corresponding tweet. Due to the ambiguity of locations of tweet posters, we use the geocoding APIs, Geopy and ArcGis Geocoding to regularise locations associated with tweets. For example, a user input location *Moselle* is transformed to a more precise and machine-parsable location: *Mosselle, Lorraine, France*. Based on the regularised locations, we filter the downloaded tweets and remove those posted by users out of GR. In total, we obtain 144,961 tweets from 8872 GR users.
- **Step 2. Social graph construction.** We construct the social graph of a large number of GR users at this step. We use an iterative approach to gradually enrich the social network. For each seed user, we obtain his/her followers and only retain those who have a mutual following relation with the seed user, because such users are more likely to reside in GR. We then download new users’ locations from their profile data and only add users from GR to the social graph. We also add edges if users in the graph have the following relation with the newly added users. After the first round, we continue going through the newly added users by adding their mutually followed friends that do not exist in the current social graph. This process will continue until no new users can be added. In our collection, it takes five iterations before termination. We take the largest weakly connected component of the social graph. After this step, we have a total of 12,256,152 users and 21,203,130 following relationships. Since the majority of users in the graph are relatively inactive, we construct a subgraph by removing all users who post or retweet less than three tweets. Note that we keep some such inactive users when the remaining graph is no longer connected after the removal of these users. In the end, we obtain a social graph with 14,756 users and 148,647 edges.
- **Step 3. Timeline tweet crawling.** In this step, we collect tweets originally posted or re-tweeted during the research period by the users in the social graph. These tweets will be used to verify the existence of info-spillover effect of users’ exposed messages on their decision to retweet information related to preventive measures, and to conduct cascade prediction experiments. Note that the tweets collected in this step are not limited to tweets related to COVID-19. In detail, we collect tweets with the newly released Twitter Academic API, which allows for downloading up to 500 tweets per user per month. We collect 18,523,099 tweets from all the users in the social graph between 22 January and 18 July 2020, covering the pandemic’s initial wave. We divide the tweets into COVID-19 related and COVID-19 unrelated based on the keywords provided by Chen et al. [30]. In our collected tweets, the COVID-19 related tweets account for 26.19%.

### 4.2. Cascade Construction and Experiment Data Selection

We construct cascades from our tweet dataset and the social graph built previously based on the definition in Section 3.1. A total of 7,485,895 cascades are built and we remove those cascades with fewer than three users, the same as the existing works [8,19]. Eventually, 89.14% of the cascades are kept and we end up with 6,672,926 cascades. The average size of these cascades is 4.31. We use C to denote the set of all the selected cascades. From C, we construct the set of cascades corresponding to messages related to preventive measures, denoted by $C_{\mathrm{PM}}$, based on the keywords listed in Table 1.

## 5. Spillover Effects in COVID-19 Preventive Measure Information Diffusion

In this section, we validate our hypothesis that the information exposed to a user has spillover effects on his/her behaviour of retweeting a message related to COVID-19 preventive measures. We first briefly describe the measurement used for quantifying the hypothesised info-exposure spillover effect. Then, we give the detailed experimental analysis designed to validate its existence in the diffusion of COVID-19 preventive measure-related messages.

### 5.1. Measuring Info-Exposure Spillover Effect

We design our validation based on the experimental investigation method commonly used for spillover effect validation [9,31]. The idea is to investigate whether users exposed to different information will behave differently in retweeting a message related to preventive measures. In other words, we will check whether certain exposed information will change the likelihood that users retweet messages related to preventive measures.

**Info-exposure spillover effect validation framework** We construct groups of users according to the information they are exposed to. Each group is composed of users who are exposed to a certain composition of information. One of these groups is set as the control group. The selection of the control group depends on the purpose of the experiment. The proportion of users in each group retweeting preventive measure messages is used to measure the likelihood of adopting preventive measure messages, which we call the *adoption likelihood*. By comparing the measurement of a group with that of the control group, we can then quantitatively evaluate the magnitude of the info-exposure spillover effect of the information exposed to this user group on adopting preventive measure messages, which we call the *info-exposure spillover elasticity*.

Formally, let D be a set of groups of nodes in G, i.e., $D=\{V_{1},\ldots ,V_{n}\}$ where $\forall_{V_{i}\in D}V_{i}\subset V$. Suppose $V_{c}\in D$ is the selected control group. For each user group $V_{i}\in D$, we identify the users who ever retweeted at least one preventive measure message in $M_{\mathrm{PM}}$, and then construct the set of identified users $V_{i}^{\mathrm{PM}}$. The *adoption likelihood* for users in $V_{i}$ is calculated as

$$\alpha_{V_{i}}=\frac{|V_{i}^{\mathrm{PM}}|}{|V_{i}|}.$$

With these notations, we can define the info-exposure spillover elasticity as follows:

**Definition** **2** (Info-exposure spillover elasticity). *The* lasticity *of the info-exposure spillover effect of a user group $V_{i}$ in the user group set D is calculated as*

$$\varepsilon_{V_{i}}^{D}=\frac{\alpha_{V_{i}}-\alpha_{V_{c}}}{\alpha_{V_{c}}}.$$

Positive elasticity indicates that the information commonly exposed to the users in $V_{i}$ increases the likelihood of retweeting a preventive measure message while negative elasticity indicates the opposite.

### 5.2. Experimental Validation of Info-Exposure Spillover Effect

We verify through our collected data that being exposed to certain information may affect users’ behaviour of retweeting messages related to preventive measures. It is not tractable to analyse all the contents that are mentioned or discussed in tweets. Therefore, inspired by previous research [32,33], we classify tweets from the level of topics and select six frequently studied ones in the literature [32,33] as the representatives. Among these topics, three are related to COVID-19, i.e., *Unemployment*, *Panic buying* and *School closures*, while the other three studied in previous Twitter-based studies are general and not directly related to the pandemic, namely, *Stop Asian hate*, *Black life matters* and *Loneliness* [34,35]. We extract corresponding tweets in each topic with the keywords listed in Table 1. According to our manual check, the keywords ensure a good coverage rate of the tweets in the selected topics. In total, the messages covered by these topics take up 18.17% of our collected tweets excluding those related to preventive measure.

For the purpose of being comprehensive, we conduct our experimental validation from two perspectives. We first evaluate the spillover effect of messages of a single topic on the behaviour of retweeting a preventive measure message. Second, we investigate the spillover effect of messages in various compositions of topics.

**Spillover effects of information of single topics** We build six sets of user groups each of which corresponds to a selected topic, i.e., $D_{U}$, $D_{\mathrm{PB}}$, $D_{\mathrm{SC}}$, $D_{\mathrm{SAH}}$, $D_{\mathrm{BLM}}$, $D_{L}$. Each set has only two groups. One consists of users that have been exposed to messages of the corresponding topic while the other group is composed of users who have not been exposed. We will take the one unexposed to the topic as the control group. In Table 2, we show the number of users exposed and unexposed in each group set, the adoption likelihood and the final info-exposure spillover effect elasticity.

We have three main observations. First, the exposure to each topic of messages will increase the likelihood of users to retweet a preventive measure message. On average, the adoption likelihood of exposed groups equals 0.58, while the unexposed group only has an activation likelihood of 0.28. The average elasticity is 1.19, which indicates that the activation likelihood doubles for the users exposed to the topics on average. Second, the increase of adoption likelihood for exposed users differs among the topics of exposed information. For instance, the exposure to information related to *Panic buying* and *Black life matters* just increases the elasticity by 0.25 and 0.16, respectively, which are much smaller than the other topics. We manually examine messages in the topic *Black life matters* and *Stop Asian hate* to understand the difference. We notice that users exposed to the messages about racists have more diverse attitudes towards prevention measures. This is consistent with previous studies [36]. For example, some users argue that the protest breaks the social distancing policy and exacerbates the virus transmission, while some others hold the view that the impact of COVID-19 is overstated and the lockdown policy worsens racial discrimination. The above two observations apply in both COVID related topics and COVID unrelated topics. Third, exposure to messages unrelated to COVID imposes a weaker spillover effect than those related to COVID. On average, the average elasticity of the COVID-19 unrelated topics is 14.76% smaller than that of the COVID-19 related topics.

From the above analysis, we can conclude that (i) exposure to certain topics of information, regardless of whether they are related to COVID-19, will impose positive spillover effects on users’ likelihood to retweet preventive measure messages; and (ii) the scale of spillover effect differs according to the topics of exposed messages.

**Spillover effects of information of compositions of topics.** In the previous analysis, we focus on the spillover effect of single topics and ignore the changes when multiple topics of information are exposed to users simultaneously. We construct a user group set $D_{\mathrm{comp}}$ of 22 groups, of which 15 groups correspond to the users who are only exposed to messages of every pair of the six topics, and six are composed of users only exposed to tweets of one of the selected topics. The last group contains the users exposed to no messages in all the topics and is chosen as the control group. Note that we do not consider the compositions of more than two topics in $D_{\mathrm{comp}}$ because we observe in our analysis that exposure to messages of any three topics leads to an adoption likelihood of at least 0.79. This indicates the improvement of an info-spillover effect will be marginal when users are exposed to messages of more topics.

**Figure 1:**
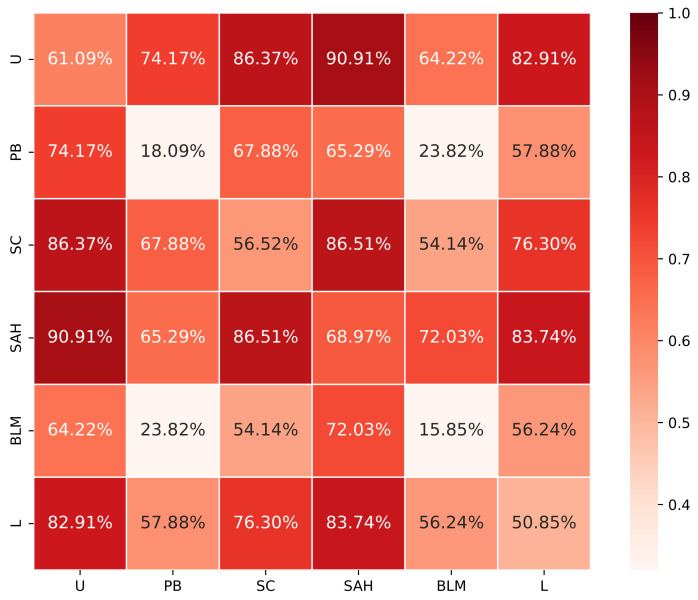
Activation likelihood when exposed to compositions of topics.

Figure 1 shows the adoption likelihood of user groups exposed to the topic compositions in $D_{\mathrm{comp}}$ except for the control group. We can see that exposure to more selected topics increases the likelihood of retweeting a preventive measure message. Exposure to an additional topic significantly increases the adoption likelihood. The most significant increase occurs to the topic of *Panic buying*. The addition of any other topic except for the topic BLM increases the adoption likelihood by at least two times. When exposed to none of the topics, the activation likelihood for the users drops below 5%.

**Discussion** From the above analysis, we empirically validated the existence of the info-exposure spillover effects. Specifically, certain information exposed to users indeed increases the likelihood of users to retweet preventive measure messages. In addition, we also illustrated that the magnitudes of this spillover effect depend on the content of tweets exposed. In the following, we will leverage deep learning to automatically capture the contents of tweets exposed to users that impose strong info-exposure spillover effects, and thus improve the accuracy of cascade prediction.

## 6. Predicting Popularity of COVID-19 Preventive Measure Messages with Spillover Effects

We use the framework of Graph Neural Networks (GNN) to learn the magnitudes of the info-exposure spillover effect of a user’s exposed information on his/her behaviour of retweeting preventive measure messages. Recall that the information exposed to a user comes from two sources: the messages posted by their friends and his/her own posts. We need to combine these two sources in a specific manner and calculate an overall representation for each user that can be used in the following cascade prediction. This explains our selection of GNN. When a user’s past posts are encoded as a vector and attached to the corresponding node as node attributes, the message passing scheme of GNN will conduct the combination. The combination may even involve the messages from users that are not incident but within a certain number of hops. In this section, we describe how we calculate nodes’ attributes with the encoding of users’ past posts, and then detail how we extend various GNN-based models to integrate the identified info-exposure spillover effect into cascade prediction.

### 6.1. Calculating Initial Node Attributes

Given a cascade of *m* at time *t*, i.e., $C_{m}^{t}$, we calculate the initial attribute of a node v∈V, denoted by $h_{v}^{0}$, by concatenating the following three components:

1. the representation vector of the messages posted by the user before t, denoted by $\delta_{v}$;
2. the activation status of the user according to the given cascade $C_{m}^{t}$, denoted by $s_{v}$;
3. the node embedding of the user’s corresponding node in the network, denoted by $e_{v}$.

Formally, we have $h_{v}^{0}=s_{v}∥\delta_{v}∥e_{v}$, where ·∥· is the concatenation operator.

**Past message encoding $\delta_{v}$** For each user *v*, we collect her/his past messages posted or retweeted before *t*. We have learnt in Section 5 that exposure to COVID related messages may impose stronger spillover effects than those unrelated to COVID. We distinguish these two types of information to capture the difference. For each type, we collect the last λ textual messages before *t* in $M_{v}$, and thus construct two lists of messages ordered by their posting time, i.e., $\left(m_{1}^{\mathrm{rel}},m_{2}^{\mathrm{rel}},\ldots ,m_{\lambda }^{\mathrm{rel}}\right)$ and $\left(m_{1}^{\mathrm{unrel}},m_{2}^{\mathrm{unrel}},\ldots ,m_{\lambda }^{\mathrm{unrel}}\right)$ for the COVID related and unrelated, respectively. Note that λ is a pre-defined hyper-parameter that should be tuned manually. RoBERTa [37] is a language pre-trained transformer to encode short texts in multiple languages into a vector of real numbers with a pre-defined length. In this paper, we use a widely used multilingual pre-trained RoBERTa variant: XLM-RoBERTa [38]. For each message, we calculate its embedding with the default XLM-RoBERTa model and obtain the corresponding lists of message representation vectors. The resulted lists are represented as $\left(z_{1}^{\mathrm{rel}},z_{2}^{\mathrm{rel}},\ldots ,z_{\lambda }^{\mathrm{rel}}\right)$ and $\left(z_{1}^{\mathrm{unrel}},z_{2}^{\mathrm{unrel}},\ldots ,z_{\lambda }^{\mathrm{unrel}}\right)$.

Many methods exist to combine these embeddings and obtain $\delta_{v}$ while considering their relative temporal importance, e.g., Hawkes process and Gated Recurrent Unit (GRU). In this paper, according to our experimental evaluation (see Section 7.4), we adopt the content-aware temporal encoding (TE) which assigns fixed importance to messages based on their temporal order. Formally,

$$\begin{array}{cc} \phi_{v}^{\mathrm{rel}} & =\sum_{i\leq \lambda }a_{i}\cdot z_{i}^{\mathrm{rel}},\\ \phi_{v}^{\mathrm{unrel}} & =\sum_{i\leq \lambda }a_{i}\cdot z_{i}^{\mathrm{unrel}}. \end{array}$$

Note that the messages related to COVID and those unrelated share the same temporal importance settings. According to our manual probe, using two different importance settings does not give notable improvement, and increases the model complexity.

In order to capture the different contributions of messages related to COVID and those unrelated, we introduce a weight parameter ρ (0≤ρ≤1) and compute the integrated past message embedding $\delta_{v}$ as follows:

$$\delta_{v}=\phi_{v}^{\mathrm{rel}}\cdot \rho +\phi_{v}^{\mathrm{unrel}}\cdot \left(1-\rho \right).$$

**Activation status $s_{v}$ & Node embedding $e_{v}$** The user activation status $s_{v}$ is set to 1 if $v\in C_{m}^{t}$ and 0, otherwise. The node embedding captures the structural properties of the user’s neighbourhoods in the graph. Following existing studies [8,19], we use DeepWalk without further fine-tuning to learn the structural embedding for each user.

### 6.2. Instantiating GNNs with the Info-Exposure Spillover Effect

We implement three variants of GNNs to integrate the info-exposure spillover effect we identified in the previous section, i.e., Graph Convolutional Networks (GCN) [39], Graph Attention Network [40] and CoupledGNN [8]. GCN is a semi-supervised learning algorithm for graph representation and GAT is a variant of GCN which introduces the attention mechanism to distinguish the significance of neighbours. These two variants are not designed specifically for cascade prediction. The calculated node representations are usually used for the downstream tasks such as link prediction and node classification. CoupledGNN [8] is a model developed for cascade prediction, and can stand for the state-of-the-art. It has overwhelming performance over existing models by simulating the cascading effect of information diffusion on social network, the phenomenon in which users are activated due to the influence from their activated neighbours. By extending these models, our purpose is to illustrate the effectiveness of info-exposure spillover effects in improving the accuracy of the predicted popularity of COVID-19 preventive measure messages. In addition, our extended models can provide useful references for future cascade prediction models to integrate info-exposure spillover effects.

The definitions of the function Aggregate(∗) and Combine(∗) of GCN, GAT and CoupledGNN are briefly given in Table 3. GAT and GCN share the same combination function. For GCN, we use the mean of the representation vectors of both the nodes and their one-hop neighbours as the aggregated value at each layer while GAT uses the weighted average.

We describe CoupledGNN in more detail due to its relatively large difference from the conventional GNN framework and explain how to simulate the cascading effect in information diffusion. For the full description, we refer the readers to the original paper [8]. It deploys two GNNs. One GNN captures the activation statuses of users during the information diffusion at each layer, e.g., the activation status of user *v* at the *ℓ*-th layer $s_{v}^{\ell }$. The other GNN aims to simulate how the influence of users changes along with the activation status and the influences of their neighbours, i.e., $r_{u}^{\ell }$. A neighbour *u*’s influence to activate user *v* in the next layer ℓ+1 is calculated by the function $\mathrm{influGate}(r_{u}^{\ell },r_{v}^{\ell })$. Then, the aggregation function is the weighted average of all the neighbours’ activation statuses with the default activation probability $p_{v}$ added. The combination function is based on the weighted average of its status on the previous layer and the aggregated representation. With the activation status output by the last layer (e.g., *k*), the popularity of the message diffused in $C_{t}^{m}$ is calculated as ${\tilde{n}}_{\infty }^{m}=\sum_{v\in V}s_{v}.$ In the following, we will describe how we extend each selected model to capture the info-exposure spillover effect.

**SE-GCN & SE-GAT.** We can interpret the output of the *k*-th layer of a *k*-layered GCN or GAT as the summary of the information exposed to every user. Then, we use an activation function to capture the info-exposure spillover effect. Specifically, the function takes as input the output of the GCN or GAT and the representation of the message diffused in the given cascade, and outputs the predicted final activation statuses of the nodes. Let *m* be the message being diffused and $z_{m}$ be the embedding vector of *m* calculated by the RoBERTa model. Let ${\tilde{s}}_{v}^{\infty }$ be the predicted activation status of node *v*. Our activation function is defined as:

$${\tilde{s}}_{v}^{\infty }=\left\{\begin{array}{cc} \mathrm{activate}\left(W_{h}h_{v}^{k}∥W_{z}z_{m}\right) & v\notin C_{m}^{t}\\ 1 & v\in C_{m}^{t} \end{array}\right.$$

where function activate is implemented as a 3-layer neural network in this paper and $W_{h}$ and $W_{z}$ are two matrices to be learned. We add this function as a downstream component after the last layer of the GCN and GAT.

**SE-CGNN.** Recall that CoupledGNN uses the function InfluGate to simulate the process of a user to be activated by their neighbours. The influence vector, e.g., $r_{u}$ of user *u*, contains user *u*’s posted messages and the messages from *u*’s neighbourhood. Therefore, it can be considered as a summary of the information perceived by a user *v* from *u* if *v* follows *u* in Twitter. Based on this intuition, we extend CoupledGNN by reformulating the function InfluGate(∗) to capture the the info-exposure spillover effect:

$$\mathrm{influGate}\left(r_{u}^{\ell },r_{v}^{\ell }\right)=\beta^{\ell }\left[W^{\ell }r_{u}^{\ell }∥W^{\ell }r_{v}^{\ell }∥W_{z}z_{m}\right].$$

### 6.3. Objective Function

We use the same objective function as [8] which is the mean relative square error (MRSE). Let $M_{C}$ be the set of diffused messages corresponding to the cascades in C whose final sizes are to be predicted. Then, MRSE can be defined as follows:

$$\begin{array}{cc} L_{\mathrm{MRSE}} & =\frac{1}{|\;M_{C}\;|}\sum_{m\in M_{C}}{\left(\frac{{\tilde{n}}_{\infty }^{m}-n_{\infty }^{m}}{n_{\infty }^{m}}\right)}^{2} \end{array}$$

This loss function is regularised to avoid over-fitting and accelerate the convergence speed, i.e., $L=L_{\mathrm{MRSE}}+L_{\mathrm{Reg}}$, where $L_{\mathrm{Reg}}=\theta \sum_{p\in P}{∥p∥}_{2}+\lambda L_{\mathrm{user}}$. Note that P denotes the set of parameters and $L_{\mathrm{user}}$ is the cross-entropy

$$\begin{array}{cc} & \frac{1}{|M_{C}|}\sum_{m\in M_{C}}\frac{1}{|V|}\sum_{v\in V}\left(s_{v,m}^{\infty }\log{\tilde{s}}_{v,m}^{\infty }+\left(1-s_{v,m}^{\infty }\right)\log\left(1-{\tilde{s}}_{v,m}^{\infty }\right)\right) \end{array}$$

where $s_{v,m}^{\infty }$ is the final activation status of *v* in the cascade of message *m* and ${\tilde{s}}_{v,m}^{\infty }$ is *v*’s status predicted by the model under evaluation.

### 6.4. Computational Complexity

In general, all our extended models inherit the complexity of the original models. According to a recent survey, the theoretical computation complexity of the message passing schemes such as GCN [39] and GAT [40] is O(|E|) [41], where |E| is the number of edges of the graph G. This is because, in these methods, the computation of each node *v*’s representation involves messages that come from its adjacent nodes. The models that are based on GCN and GAT, proposed previously, i.e., SE-GCN and SE-GAT, also work in the same way, and thus have the complexity of O(|E|). Similarly, SE-CGNN has the same computational complexity as CGNN, i.e., O(p|V|+q|E|) [8], where *p* and *q* are the constants determined by the batch sizes, and |V| is the number of nodes in G.

## 7. Experimental Evaluation

### 7.1. Evaluation Measurements

We adopt the measurements in [8] to evaluate and compare the prediction performance of our extended models and the bench-markings models in our experiments. Specifically, in addition to the mean relative square error (MRSE) introduced in the previous section, we also use mean absolute percentage error (MAPE) and wrong percentage error (WroPerc). MAPE measures the average deviation between the predicted popularity and the true values, while WroPerc measures the percentage of cascades that are incorrectly predicted with a given error tolerance ϵ. Formally, they can be defined as follows:

$$\begin{array}{cc} \mathrm{MAPE} & =\frac{1}{|M_{C}|}\sum_{m\in M_{C}}\frac{|{\tilde{n}}_{\infty }^{m}-n_{\infty }^{m}|}{n_{\infty }^{m}},\\ \mathrm{WroPerc} & =\frac{1}{|M_{C}|}\sum_{m\in M_{C}}I\left[\frac{|{\tilde{n}}_{\infty }^{m}-n_{\infty }^{m}|}{n_{\infty }^{m}}\geq \varepsilon \right]. \end{array}$$

Note that I(∗) is an indication function which outputs 1 when the input proposition is true or 0 otherwise, and the threshold ε is set as 0.5 in our experiments. For all the three measurements, smaller values indicate better performance.

### 7.2. Baseline Methods

In addition to CoupledGNN, we use the following models as baselines.

- **Feature-based method**. This is a linear regression model with L2 regularisation with features. For better comparison, we adopt the same features used in the past studies [8,19].
- **SEISMIC** [18]. SEISMIC uses the Hawkes self-activation point process to estimate or approximate the impact of cascading effect with their average number of followers.
- **DeepCas** [19]. DeepCas is an end-to-end deep learning method for information cascades’ prediction. It utilises the structure of the cascade graphs for prediction. An attention mechanism is designed to assemble a cascade graph representation from a set of random walk paths.
- **DeepHawkes** [20]. DeepHawkes is also an end-to-end deep learning method for information cascades prediction. It combines user embedding vectors and cascades encoding by RNNs, and then uses the Hawkes process to model and predict information cascade.
- **CasCN** [22]. CasCN for cascade modelling and prediction is achieved by splitting the cascade graph into a series of sequential sub-cascades and then employing GCN to learn the structural information of the cascades.
- **GCN and GAT**. We construct these two models from our SE-GCN and SE-GAT models by removing the representation vectors of messages. In other words, these two models only rely on network structure to predict the sizes of final cascades.

We implement several variants of our extended models, i.e., SE-GCN, SE-GAT and SE-CGNN according to the methods used to integrate users’ past messages with their temporal significance considered. We consider three other methods in addition to the TE methods adopted in our model, namely, Mean, Hawkes and GRU. Note that, regarding Hawkes and GRU, we use their basic versions. The method Mean calculates the average embedding vectors of the past messages for both $\phi_{v}^{\mathrm{rel}}$ and $\phi_{v}^{\mathrm{unrel}}$. In order to distinguish these variants, we append the corresponding methods at the end of the model names. For instance, SE-CGNN-TE corresponds to the implementation of the model presented in Section 6, and SE-CGNN-Hawkes replaces the TE method in SE-CGNN-TE with the Hawkes process.

### 7.3. Implementation Details

As the output of the RoBERTa for a sentence is a high-dimensional and sparse vector, we apply linear transformation to map its output to a relatively low-dimensional space. The dimension of the final text embedding used is set as 128. For all models including the bench-marking models, we tune their hyper-parameters to guarantee their performance on validation sets. The L2-coefficients are chosen from $0.5,0.1,0.05,\cdots ,10^{-8}$. For all neural network models, the learning rate is chosen from $0.1,0.05,\cdots ,10^{-5}$. The coefficient in the loss function is set to be 0.5, and the mini-batch size is chosen from 15, 10, 5. The number of GNN layers *k* is selected from 5, 4, 3, 2. As for DeepCas, the number of walk sequences and the walk length are set as 100 and 8, respectively. For SEISMIC, we follow the parameters from the original study. Moreover, we randomly select 80%, 10%, 10% of the set of cascade instances for training, validation and testing, respectively.

**Figure 2:**
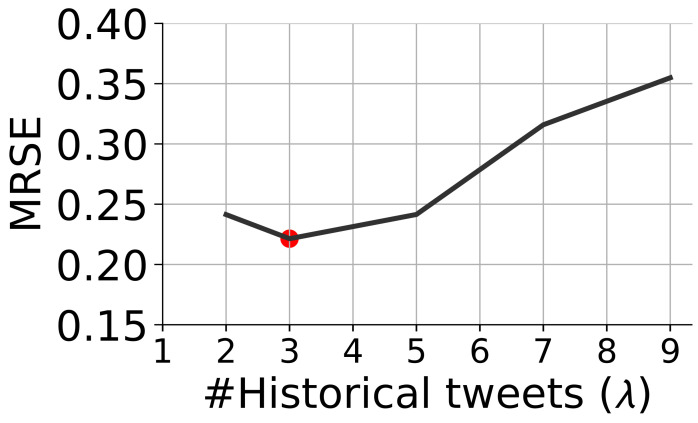
Parameter tuning for λ.

Considering the diffusion time of the messages in our collected data, we set the observation time window as three hours and construct the set of observed cascades, i.e., C, by removing users in our cascades that were activated after the first three hours. The number of past messages λ is critical in enforcing the quality of prediction. As a result, we undertake an empirical investigation to identify the impact of λ on the final performance. We present the MRSE with different values of λ when the SE-CGNN-TE is used in Figure 2. We observe that λ does have an important impact on prediction results. We set λ as 3 with which our model achieves the best performance.

As we repeatedly emphasised, our original goal is to predict the popularity of messages on social media which are related to COVID preventive measures. In order to comprehensively evaluate the effectiveness of the info-exposure spillover effect, in addition to the cascades of preventive measure messages $C_{\mathrm{PM}}$, we also apply all the models on another two sets of cascades. One is the set of all cascades C. The other is the set of cascades that are not related to preventive measures, i.e., ${\bar{C}}_{\mathrm{PM}}=C/C_{\mathrm{PM}}$, the complement of $C_{\mathrm{PM}}$ in C.

### 7.4. Experimental Results

We show the performance of all the above-mentioned models in Table 4 in the form of the three selected measurements. In general, we can observe three obvious differences when the info-exposure spillover effect is introduced in cascade prediction.

First, compared to the original models, our extended models significantly improve their performance not only for the preventive measure messages, but also for all three types of messages. The most significant improvement occurs to SE-CGNN-TE and reaches 23% in the WroPerc measurement for the preventive measure messages and over 10% for the messages unrelated to preventive measures. This is due to the fact that CoupledGNN simulates the cascading effects iteratively, and this allows for applying the info-exposure spillover effect on activating individual users in a finer granularity. From the above analysis, we can conclude that the use of the info-exposure spillover effect can effectively improve the performance of existing cascade prediction models. It should be integrated into future models by design.

Second, we observe that the extended models can more accurately predict the popularity of COVID-19 preventive measure messages than the other messages, which is the opposite for the baseline models. For the baseline models, their performance on C and ${\bar{C}}_{\mathrm{PM}}$ are almost the same but becomes worse on $C_{\mathrm{PM}}$. The feature-based model has the worst performance which decreases by over 11% compared to that in predicting the sizes of the other two sets of cascades. However, when the identified info-exposure effect is used in our extended models, the popularity of preventive measure messages can be predicted with better accuracy. SE-CGNN-TE can improve the performance by about 13.8% for preventive measurement messages compared to those unrelated to preventive measures. This observation validated empirically that the exposure to information generated during the COVID-19 pandemic has strong spillover effects on retweeting messages about how to prevent the transmission of the COVID virus.

Third, the consideration of the temporal importance of past tweets does bring about further improvement, and our selected TE method overwhelms the other widely used ones. The method Mean which ignores the temporal significance of past messages produces the worst predictions. Hawkes and GRU have similar performances. Compared to them, our TE method leads to an improvement of about 0.02 in all three types of cascades.

### 7.5. Compare SE-CGNN-TE with Its Variants

Recall that we distinguish the messages related to COVID-19 and those unrelated in integrating the embedding vectors of users’ past messages into the initial node attributes (see Section 6.1). We use a parameter ρ to learn the relative importance of the message related to COVID-19. We conduct additional experiments to justify our selection. Specifically, we implement another three variants of our SE-CGNN-TE model. The first one, named SE-CGNN-TE-REL, only takes the last λ messages that are related to COVID-19 as a user’s past messages. Similarly, the second variant, named by SE-CGNN-TE-UNREL, only consider those unrelated to COVID-19. The last SE-CGNN-TE-ALL variant ignores the difference and straightforwardly consider the last λ messages regardless of their types. The same as our previous experiments, we train these three variants and run them on the three sets of testing cascades, i.e., C, $C_{\mathrm{PM}}$, and ${\bar{C}}_{\mathrm{PM}}$. The results are shown in Table 5. We also include the results of SE-CGNN-TE for comparison.

In general, we have two main observations. First, we observe that, among the three variants, the one with only messages related to COVID generates the best performance while the one only utilising those unrelated to COVID performs the worst. This also confirms our findings in Section 5.2 that COVID related messages tend to impose stronger spillover effects on retweeting preventive measure messages. This performance difference also indicates that this finding may also apply to other messages which are not relevant to preventive measures. Second, the integration method used in SE-CGNN-TE can effectively improve the performance. This improvement may come from two sources. On one hand, our selected method actually uses 2λ past messages. This implies that more information can help increase the prediction accuracy. On the other hand, a balance between these two types of information can be reached during the model training.

## 8. Discussion and Conclusions

In this paper, we concentrated on the problem of cascade prediction for COVID-19 information about preventive measures on online social media platforms. Compared to previous works, we took into account the phenomenon that the exposure to different information will influence social media users’ behaviour of participating in information diffusion during the pandemic, which we call *info-exposure spillover effect*. With a dataset we collected from Twitter, we successfully validated its existence. In particular, both COVID-19 related and unrelated messages may have spillover effects on the spread of COVID-19 messages promoting preventive measures. Meanwhile, the COVID related messages tend to impose stronger spillover effects. We then applied the identified spillover effects in predicting the popularity of preventive measure messages. Specifically, we built three new models by making use of the recent advances of graph representation techniques, i.e., graph neural networks (GNN). In addition, we utilised a temporal encoding method to capture the important variance caused by message posting time. With extensive experiments, we showed that our new models outperform baselines not only for preventive measure messages but for all messages. This illustrates that the use of info-exposure spillover effect can effectively improve the performance of cascade prediction, and it should be recommended to be considered in designing future cascade prediction models. Specifically, we through this paper showcased a general method that can be referred to, in order to validate the existence of spillover effects of other types of information on the changes of information consumers’ behaviours which are not restricted to retweeting. Moreover, other applications can also benefit from our work. For instance, social media posts have been used to extract effective indicators, e.g., numbers of daily posts and their sentiments [42], in predicting the price of cryptocurrencies such as Bitcoin. Our extended models can help accurately forecast the popularity of Bitcoin influencers’ social media posts, e.g., Elon Musk [43], which can be integrated into existing models to further improve the accuracy of predicted prices. As our future work, we will consider other types of information in addition to users’ textual posts and propose new methods to integrate them in cascade prediction.

We identified three main limitations that have not been well addressed in our current research. First, our empirical validation of the info-exposure spillover effect only focused on messages on Twitter related to preventive measures and conducted from the level of selected topics. Although in our experiment the overwhelming performance of our extended models on other general messages could partially validate its existence, finer-grained and more comprehensive analysis will be desired and we will take this as our future work. Second, our cascade prediction models are extended from existing GNN models. It will be interesting to design a new end-to-end GNN model which is specifically adapted to the identified spillover effects of users’ adopted information. Finally, we only distinguished the significant difference between messages related and unrelated to COVID while ignoring the other linguistic features of individual messages.

## Figures and Tables

**Table 1:** Keyword lists for filtering tweets related to preventive measures and selected topics.

Topic	Abbre.	Keywords
Preventive measure	PM	stayathome, mask, masque, maske, wash hand, social distancing, socialdistancing, staysafe, lockdown
Unemployment	U	job, jobsearch, unemployment, employment, career, resume, recruitment, recession, economy, economic emploi, stelle, employ, arbeitslos, chômeurs
Panic buying	PB	panicbuying, panicshopping, panicbuyers, toiletpaper, handsanitizer, coronashopping
School closures	SC	schoolclos, closenypublicschool, closenycschools, suny, cuny, homeschool, noschool, shutdownschools
Stop Asian hate	SAH	stopasianhate, stopaapihate, stopasianhatecrimes, asian, aapi, asianlivesmatter, asiansareguman, antiasianhate
Black life matters	BLM	blacklifematters, blacklivesmatter, atlantaprotest, blm, changethesystem, justiceforgeorgefloyd
Loneliness	L	lonely, loneliness, alone, solitaire, solitude, seul, einsam, einsamkeit, allein

**Table 2:** Validation of info-exposure spillover effect of single topics.

Topic Type	Topic	Exposed		Unexposed		Elasticity ε
		#User	α	#User	α	
COVID Related	Unemployment (U)	4238	0.67	17,101	0.25	1.69
	Panic buying (PB)	6119	0.39	15,220	0.31	0.25
	School closures (SC)	6460	0.61	14,879	0.21	1.87
COVID unrelated	Stop Asian hate (SAH)	6740	0.72	14,599	0.28	1.53
	Black life matters (BLM)	9041	0.48	122,98	0.41	0.16
	Loneliness (L)	5343	0.79	15,996	0.30	1.63

**Table 3:** Brief description of selected GNN variants.

Model	Aggregate(∗)	Combine(∗)
**GCN**	$a_{v}^{\ell }=\frac{\sum_{u\in N(v)\cup \{v\}}h_{u}^{\ell -1}}{|N(v)\cup \{v\}}$	$h_{v}^{\ell }=\mathrm{LeakyReLu}\left(W^{\ell }a_{v}^{\ell }\right)$
**GAT**	$\begin{array}{cc} & a_{v}^{\ell }=\sum_{u\in N(v)\cup \{v\}}\beta_{\mathrm{uv}}^{\ell }h_{u}^{\ell -1}\\ & \beta_{\mathrm{uv}}^{\ell }=\frac{\exp\left(\mathrm{LeakyRelu}(\gamma^{T}\left[{\mathrm{Wh}}_{u}^{\ell -1}∥{\mathrm{Wh}}_{v}^{\ell -1}\right])\right)}{\sum_{u^{\prime }\in N\left(v\right)\cup \left\{v\right\}}\exp\left(\mathrm{LeakyRelu}(\gamma^{T}\left[{\mathrm{Wh}}_{u^{\prime }}^{\ell -1}∥{\mathrm{Wh}}_{v}^{\ell -1}\right])\right)} \end{array}$	$h_{v}^{\ell }=\mathrm{LeakyReLu}\left(W^{\ell }a_{v}^{\ell }\right)$
**CoupledGNN**	$\begin{array}{cc} & a_{v}^{\ell }=\sum_{u\in N(v)}\mathrm{InfluGate}\left(r_{u}^{\ell -1},r_{v}^{\ell -1}s_{u}^{\ell -1}+p_{v}\right.\\ & \mathrm{influGate}\left(r_{u}^{\ell },r_{v}^{\ell }\right)=\beta^{\ell }\left[W^{\ell }r_{u}^{\ell }∥W^{\ell }r_{v}^{\ell }\right] \end{array}$	$s_{v}^{\ell +1}=\left\{\begin{array}{cc} 1 & v\in C_{m}^{T}\\ \sigma (\mu_{s}^{\ell }s_{v}^{\ell }+\mu_{a}^{\ell }a_{v}^{\ell }) & v\notin C_{m}^{T} \end{array}\right.$

**Table 4:** Cascade prediction performance of our extended models and baselines.

Models	C			$C_{\mathrm{PM}}$			${\bar{C}}_{\mathrm{PM}}$		
	MRSE	MAPE	WroPerc	MRSE	MAPE	WroPerc	MRSE	MAPE	WroPerc
Feature-based	0.3611	0.4018	41.31%	0.4403	0.4049	46.08%	0.3704	0.4151	41.56%
SEISMIC	0.5580	0.5104	56.35%	0.5899	0.5265	55.88%	0.5419	0.5083	56.14%
DeepCas	0.2837	0.3959	37.71%	0.2847	0.3724	38.67%	0.2872	0.4010	37.31%
DeepHawkes	0.3278	0.4089	37.10%	0.3297	0.4092	37.94%	0.3213	0.3948	36.78%
CasCN	0.3097	0.4300	37.12%	0.3017	0.4166	40.39%	0.3098	0.4106	37.58%
GCN	0.3144	0.4217	38.88%	0.3179	0.4238	41.76%	0.3110	0.4200	38.69%
SE-GCN-Mean	0.2826	0.4056	36.76%	0.2757	0.3990	35.86%	0.2899	0.4178	36.82%
SE-GCN-Hawkes	0.2826	0.4056	36.76%	0.2708	0.3961	35.44%	0.2887	0.4126	36.89%
SE-GCN-GRU	0.2875	0.4085	36.87%	0.2712	0.3974	35.43%	0.2871	0.4124	36.92%
SE-GCN-TE	0.2802	0.4050	36.15%	0.2702	0.3932	35.20%	0.2819	0.4109	36.15%
GAT	0.3072	0.4211	39.19%	0.3014	0.4268	40.01%	0.3101	0.438	39.85%
SE-GAT-Mean	0.2862	0.4124	37.58%	0.2721	0.4001	35.31%	0.2903	0.4175	38.64%
SE-GAT-Hawkes	0.2790	0.4078	37.45%	0.2654	0.3986	35.30%	0.29353	0.4154	37.83%
SE-GAT-GRU	0.2762	0.4055	37.05%	0.2680	0.3964	35.58%	0.2961	0.4153	37.47%
SE-GAT-TE	0.2744	0.4014	37.56%	0.2673	0.3990	35.16%	0.2896	0.4177	38.06%
CoupledGNN	0.2678	0.3861	35.19%	0.2769	0.3920	34.44%	0.2601	0.3812	34.70%
SE-CGNN-Mean	0.2414	0.3610	34.17%	0.2587	0.3801	30.13%	0.2561	0.3608	33.22%
SE-CGNN-Hawkes	0.2240	0.3432	31.10%	0.2085	0.3171	27.44%	0.2271	0.3478	31.35%
SE-CGNN-GRU	0.2283	0.3469	32.28%	0.2174	0.3164	28.65%	0.2411	0.3625	33.04%
SE-CGNN-TE	**0.2131**	**0.3358**	**30.63%**	**0.2031**	**0.3073**	**27.78%**	**0.2262**	**0.3437**	**31.56%**

**Table 5:** The performance comparison of methods of past message integration.

Models	C			$C_{\mathrm{PM}}$			${\bar{C}}_{\mathrm{PM}}$		
	MRSE	MAPE	WroPerc	MRSE	MAPE	WroPerc	MRSE	MAPE	WroPerc
SE-CGNN-TE-REL	**0.2171**	**0.3367**	**32.23%**	**0.2210**	**0.3294**	**28.46%**	**0.2312**	**0.3526**	32.96%
SE-CGNN-TE-UNREL	0.2357	0.3572	33.83%	0.2442	0.3690	30.74%	0.2484	0.3567	33.01%
SE-CGNN-TE-ALL	0.2208	0.3406	32.53%	0.2318	0.3172	28.96%	0.2470	0.3534	**32.80%**
SE-CGNN-TE	**0.2146**	**0.3351**	**30.45%**	**0.2024**	**0.3062**	**27.41%**	**0.2268**	**0.3421**	**31.20%**

## Data Availability

The data used to support the findings of this study are available from the corresponding author upon request.
